# Shikonin suppresses NEAT1 and Akt signaling in treating paclitaxel-resistant non-small cell of lung cancer

**DOI:** 10.1186/s10020-020-00152-8

**Published:** 2020-04-08

**Authors:** Farong Zang, Yuanquan Rao, Xinhai Zhu, Zhibing Wu, Hao Jiang

**Affiliations:** 1 Department of Respiratory and Oncology, Changxing County People’s Hospital, Changxing, Zhejiang 313100 People’s Republic of China; 2 Department of Oncology, Zhejiang Hospital, No.12 Lingyin Road, Hangzhou, Zhejiang 310013 People’s Republic of China

**Keywords:** Shikonin, Drug-resistance, NSCLC, NEAT1, Akt

## Abstract

**Background:**

The development of paclitaxel-resistance led to the tumor relapse and treatment failure of non-small cell lung cancer. Shikonin has been demonstrated to show anti-cancer activity in many cancer types. The present study aimed to investigate the anti-cancer activity of shikonin in paclitaxel-resistant non-small cell lung cancer treatment.

**Methods:**

MTT, clonogenic assay, apoptotic cell death analysis, western blot, qRT-PCR, gene knockdown and overexpression, xenograft experiment, immunohistochemistry were performed.

**Results:**

Shikonin decreased paclitaxel-resistant NSCLC cell viability and inhibited the growth of xenograft tumor. Shikonin induced apoptotic cell death of paclitaxel-resistant NSCLC cell lines and suppressed the level of NEAT1 and Akt signaling of paclitaxel-resistant NSCLC cell lines and xenograft tumors. Either low dose or high dose of shikonin considerably suppressed the cell growth and induced the cell apoptotic death in NEAT1 knockdown A549/PTX cells, and p-Akt expression was decreased.

**Conclusions:**

Shikonin could be a promising candidate for paclitaxel-resistant NSCLC treatment.

## Introduction

Globally, lung cancer is the most lethal malignancy and the leading cause of death. More than 85% of lung cancer patients were diagnosed as non-small cell lung cancer (NSCLC). Unfortunately, a lot of NSCLC patients are an advanced disease when the first diagnosis and unsuitable for surgery. Despite the development of immunotherapy and targeted drugs, chemotherapy remains the cornerstone of lung cancer treatment. And clinically, paclitaxel is a preferred chemotherapeutic agent and the first-line drug for advanced NSCLC (Adrianzen Herrera et al. 2019). However, the development of paclitaxel-resistance led to tumor relapse and treatment failure, which limits the application of paclitaxel. Understanding of the mechanisms of paclitaxel-resistance may improve the NSCLC treatment outcomes, but the molecular mechanisms of drug resistance are complicated. The development of drug resistance may be related to gene mutation, overactivation of by-pass signaling and aberrant expression of efflux protein. Previous studies indicated that long non-coding RNA (lncRNA) played an important role in the paclitaxel-resistance of several types of cancer. Linc01118, UCA1, Nuclear-enriched abundant transcript 1 (NEAT1) contributed paclitaxel-resistance to ovarian cancer (Shi and Wang 2018; Wang et al. 2018a; An et al. 2017), FTH1P3, H19 activated paclitaxel-resistance in breast cancer (Wang et al. 2018b; Han et al. 2018), Linc00518, Linc00473, PVT1 promoted paclitaxel-resistance of prostate cancer, colorectal cancer and glioma respectively (He et al. 2019; Wang et al. 2018c; Song et al. 2018), and NEAT1, KCNQ1OT1, ANRIL conferred paclitaxel-resistance to lung cancer (Li et al. 2019; Ren et al. 2017; Xu et al. 2017). Therefore, finding an effective and safe anti-cancer agent to overcome the paclitaxel-resistance of NSCLC and explore the underlying mechanism is necessary.

Recently, compounds derived from natural herbs have attracted attention due to their curative effect and low toxicity in cancer therapy (Jiang et al. 2014a; Jiang et al. 2014b; Zhao et al. 2015; Zhu et al. 2016; Zhao et al. 2016; Song et al. 2016; Zheng et al. 2017; Wang et al. 2017; Hong et al. 2019; Yang et al. 2018). Shikonin, a naphthoquinone, is extracted from the plant root of *Lithospermum erythrorhizon*. Shikonin has been demonstrated to treat inflammation, virus infection and tumor due to its anti-inflammatory, anti-bacterial, and anti-tumor properties (Wang et al. 2019). Shikonin inhibited cell proliferation, migration, and invasion of a variety of tumors through several possible molecular mechanisms. Furthermore, shikonin overcame multidrug resistance in many cancer types and mediated lncRNA related signaling pathways (Li et al. 2018; Li et al. 2017; Zhang et al. 2017a; Yang and Chen 2015). However, the anti-cancer activity and key target of shikonin on paclitaxel-resistant NSCLC are still unclear.

In this study, the therapeutic potentials of shikonin on paclitaxel-resistant NSCLC were investigated and the underlying mechanisms were further determined. As expected, shikonin inhibited the growth of paclitaxel-resistant NSCLC through suppression of NEAT1 and Akt signaling, providing a fascinating opportunity for paclitaxel-resistant NSCLC treatment.

## Materials and methods

### Drug, reagents and cell line

Shikonin (C_16_H_16_O_5_) was obtained from Sigma-Aldrich (MO, USA). MTT, apoptosis Detection Kit and p-Akt antibody were obtained from Sigma-Aldrich (MO, USA). A549 cells were obtained from the cell bank of Chinese Academy of Sciences (Shanghai, China). The paclitaxel-resistant cell line (A549/PTX) was established as previously described (Li et al. 2019). A549 cells were exposed to a high concentration of paclitaxel and the resistant clones were selected. The paclitaxel-resistant cells were cultured in the medium of RPMI-1640 with 10% fetal bovine serum in an incubator containing 5% CO_2_ at 37 °C.

### MTT assay

Exponentially growing A549/PTX cells were seeded in 96-well plates (5000 cells/well) and cultured overnight. Cells were incubated with different concentrations of shikonin for 24 h, 48 h, and 72 h. Then MTT solution was added and incubated in the dark for 4 h. The formazan crystal was dissolved with DMSO before detecting the absorbance at 570 nm wavelength using a multi scanner auto reader.

### Clonogenic assay

A549/PTX cells were trypsinized, washed, and seeded in 6-well plates (1000 cells/well). Following treating with shikonin for 48 h, the A549/PTX cells were further incubated with fresh medium for 2 weeks. Colonies were washed with PBS, fixed with methanol, and stained with crystal violet. Colonies were scored under a light microscope.

### Apoptosis detection

Exponentially growing A549/PTX cells were seeded in 6-well plates (20,000 cells/well) and cultured overnight. The cells were then treated with different concentrations of shikonin for 48 h. After treatment, A549/PTX cells were harvested, washed with PBS, and stained with Annexin V-FITC and PI in the dark for 15 min at room temperature. Apoptosis was detected using a flow cytometer.

### Western blot analysis

Briefly, cells were treated, then harvested and lysed. The extracted protein was quantified by BCA assay and then separated with SDS-PAGE, followed by transferring onto the PVDF membrane and blocking with 5% skimmed milk in PBS. The membrane was incubated with primary antibody against p-Akt, Akt, cleaved PARP, cleaved caspase-3(1:1000) at 4 °C overnight and secondary antibody (1:10,000) at room temperature for 1 h. Then the blots were visualized using an ECL system.

### Quantitative reverse transcription-polymerase chain reaction (qRT-PCR)

Total RNA was extracted from A549/PTX cells using TRIzol reagent. RNA was reverse-transcribed with high-capacity cDNA reverse transcription kits. The cDNA was further amplified using PCR with the specific primer as follows: NEAT1: 5′-CTTCCTCCCTTTAACTTATCCATTCAC-3′(forward), 5′-CTCTTCCTCCACCATTACCAACAATAC-3′(reverse). β-actin was used for endogenous control. qRT-PCR was performed by Applied Biosystems StepOne™ Real-Time PCR System using SYBR® Premix DimerEraser Kit. The relative expression of NEAT1 was calculated using the 2^-ΔΔCt^ method and normalized to β-actin.

### NEAT1 knockdown and overexpression for transfection

NEAT1 knockdown and NEAT1 overexpression were carried out by synthesization of shRNA that specifically targeted NEAT1 (shNEAT1) and the PCR-amplified NEAT1 (NEAT1) (Genechem co., Shanghai, China). A549/PTX cells were transfected with lentiviral plasmid according to the manufacturer’s instructions.

### Nude mice xenograft experiment

Four-week-old, male, weighing 20 ± 1 g BALB/c nude mice were used. The mice were kept in the pathogen-free conditioned facility. The animal experiment was approved by the ethics committee of Zhejiang Hospital (Hangzhou, China) according to the animal care guidelines. A549/PTX cells were trypsinized and suspended, then injected subcutaneously into the flank of mice to establish paclitaxel-resistant NSCLC xenograft(*N* = 10). The mice were intraperitoneally injected with shikonin (2 mg/kg) daily after the average volume of the tumor reached 200 mm^3^. The formula (0.5 × length × width^2^) was used to evaluate tumor growth. The mice were sacrificed after the treatment lasted for 21 days. The tumors were removed and weighed, and the samples were prepared for further analysis.

### Immunohistochemistry (IHC)

Tumor samples were fixed, embedded in paraffin, and sliced up. Then slices (4 μm) were deparaffinized and then incubated with citrate buffer for antigen retrieval. The slices were incubated with primary antibody (anti-p-Akt, 1:100) overnight at 4 °C and then the second antibody for 1 h at room temperature, followed by DAB substrate incubation. The immunostaining of p-Akt was observed by light microscopy.

### Statistical analysis

The data were presented as mean ± standard deviation and analyzed using GraphPad Prism 6 (CA, USA). One-way ANOVA with the SNK-q post hoc test was performed. *p* < 0.05 was considered a statistically significant difference.

## Results

### Shikonin decreased paclitaxel-resistant NSCLC cell viability and inhibited the growth of xenograft tumor

**Fig. 1:**
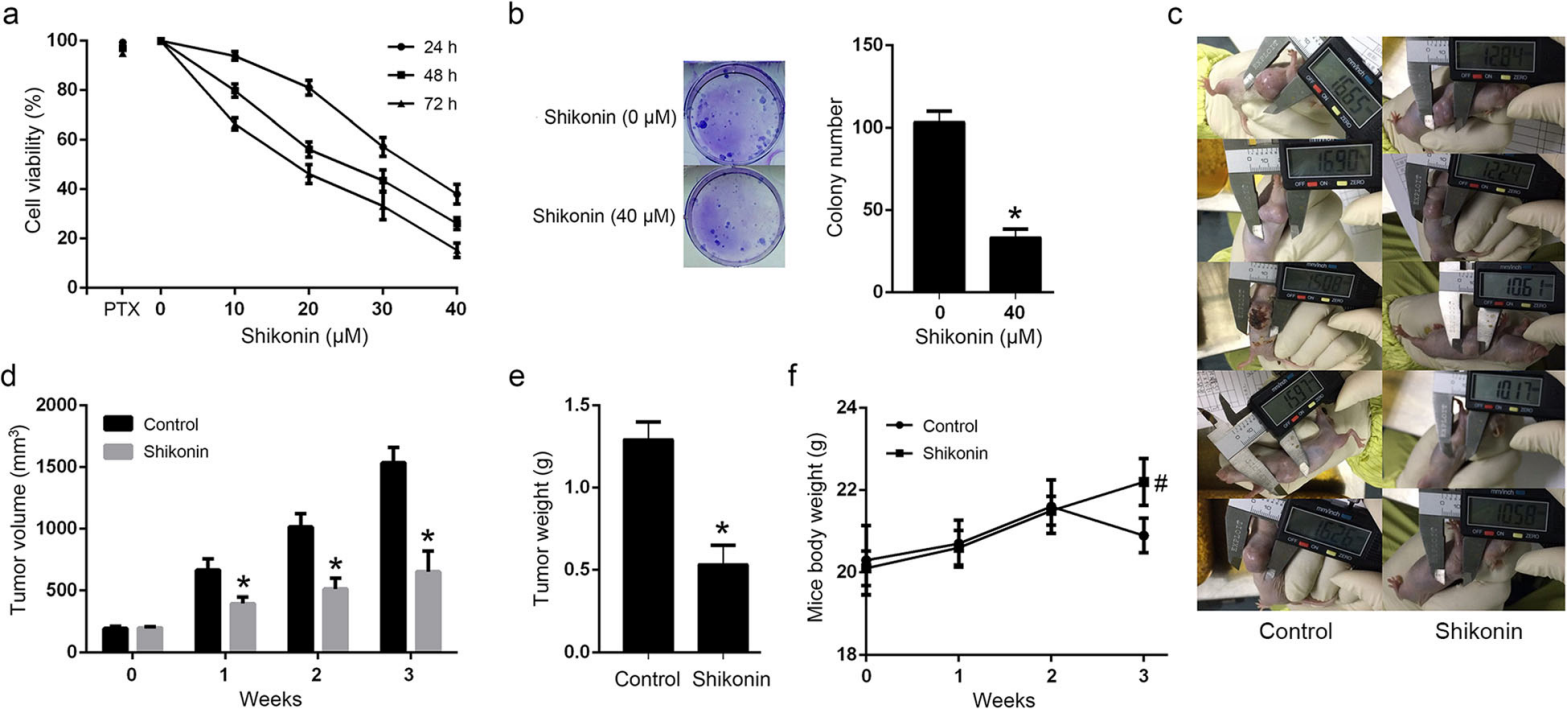
Shikonin decreased paclitaxel-resistant NSCLC cell viability and inhibited the growth of xenograft tumors. **a** Shikonin considerably decreased cell viability in A549/PTX cells in a dose- and time-dependent manner. **b** shikonin inhibited the colony formation activity of A549/PTX cells at 48 h. **c**, **d** Shikonin inhibited the tumor volume of A549/PTX xenograft. **e** Shikonin inhibited the tumor weight of A549/PTX xenograft. **f** Shikonin did not decrease the mice’s body weight. *Statistically significant difference (*p* < 0.05). #No statistically significant difference(*p* > 0.05)

A549/PTX cell line was established and used for investigating the anti-proliferative effect of shikonin in paclitaxel-resistant NSCLC. MTT assay showed that shikonin considerably decreased cell viability in A549/PTX cells in a dose- and time-dependent manner (Fig. 1a), and colony formation assay indicated that shikonin inhibited the colony formation activity of A549/PTX cells at 48 h (Fig. 1b). Further, the A549/PTX xenograft model was established to study the anti-cancer activity of shikonin in vivo. Shikonin showed significant tumor growth inhibition than the control group. Shikonin resulted in a 57.5% tumor volume decrease and 58.9% tumor weight inhibition compared to the control group, respectively (Fig. 1c, d, e). Besides, the bodyweight decrease in the shikonin-treated mice was not observed (Fig. 1f).

### Shikonin induced apoptotic cell death of paclitaxel-resistant NSCLC cell lines

**Fig. 2:**
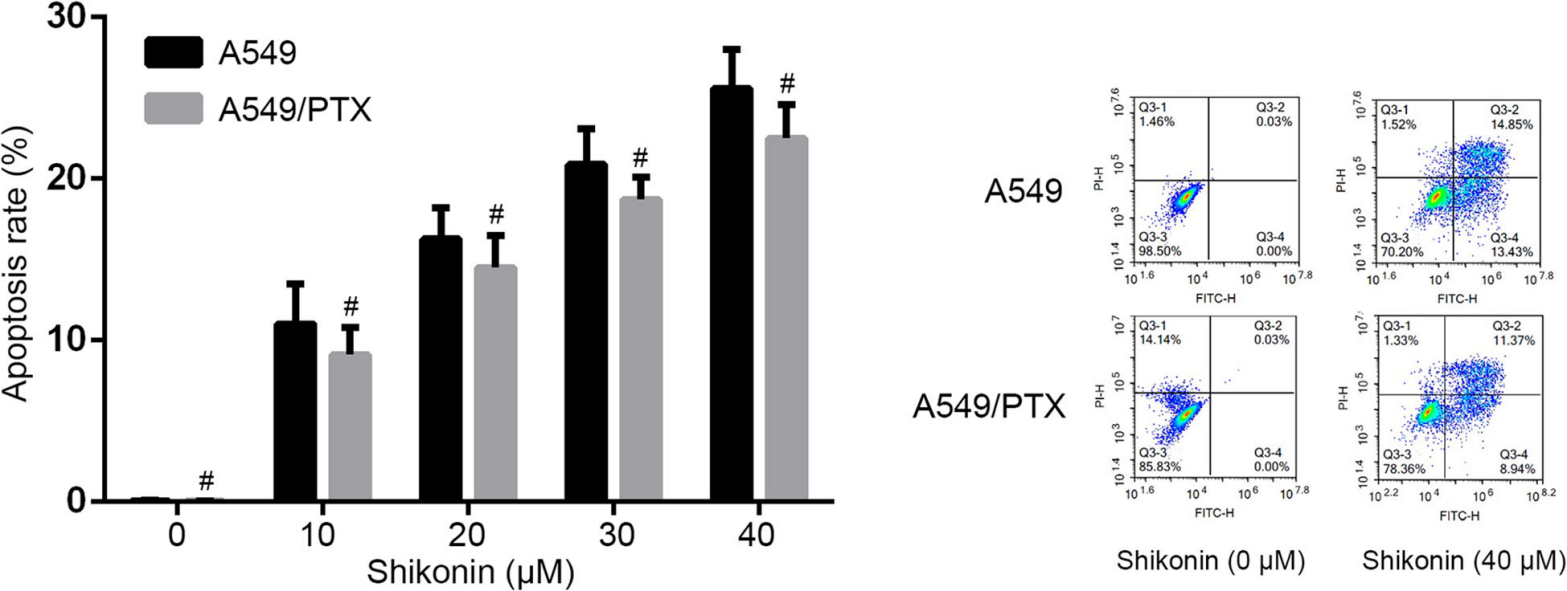
Shikonin induced apoptotic cell death of paclitaxel-resistant NSCLC cell lines in a dose-dependent manner. #No statistically significant difference(*p* > 0.05)

We investigated apoptotic cell death in paclitaxel-resistant A549/PTX cells after shikonin treatment. The cells were treated with shikonin for 48 h and then determined by Annexin V assay. We found that shikonin markedly increased A549/PTX cell apoptosis in a dose-dependent manner, and the apoptosis rate was similar to the A549 cells treated by shikonin (Fig. 2).

### Shikonin suppressed the level of NEAT1 and Akt signaling of paclitaxel-resistant NSCLC cell lines and xenograft tumor

**Fig. 3:**
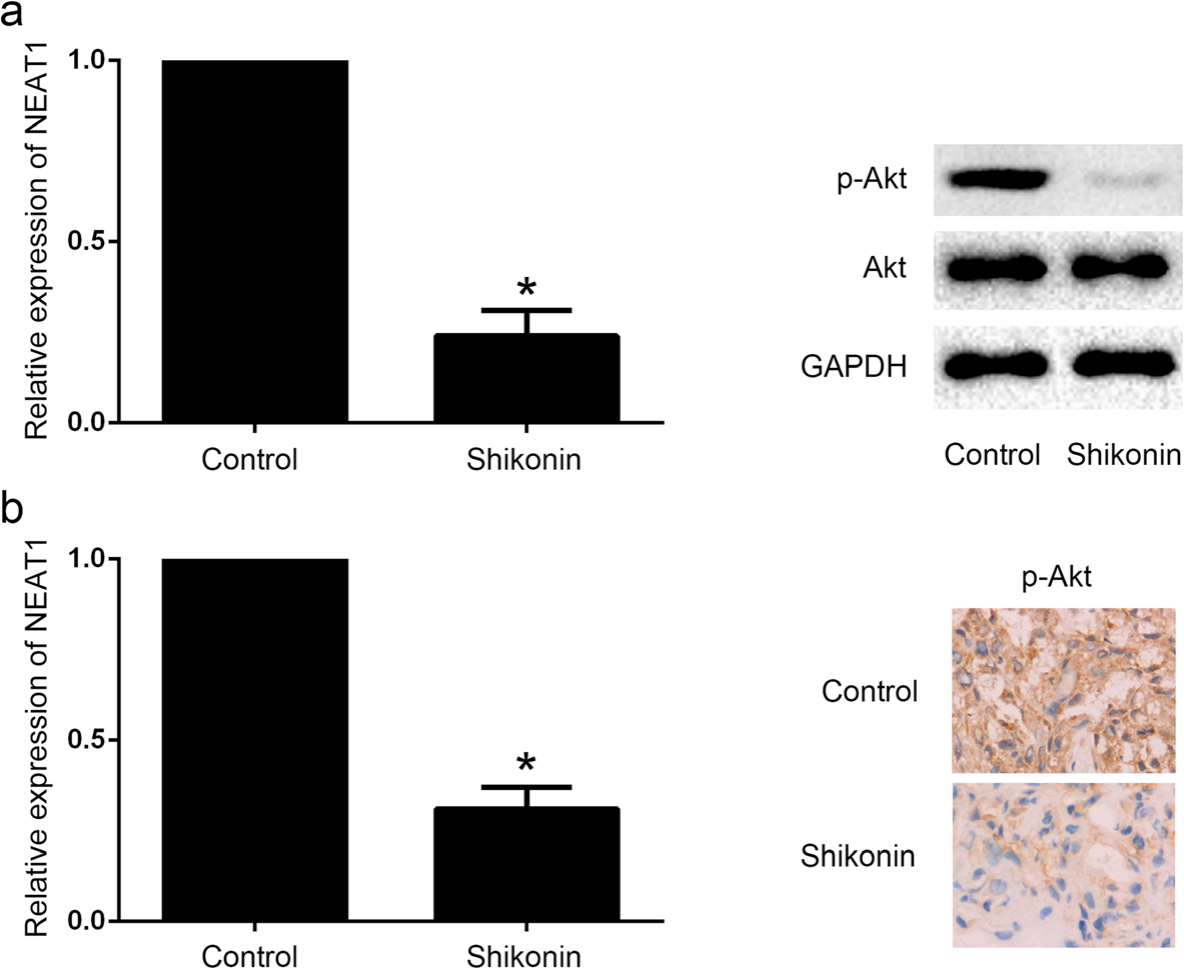
Shikonin suppressed the level of NEAT1 and Akt signaling of paclitaxel-resistant NSCLC cell lines and xenograft tumors. **a** The level of NEAT1 expression was downregulated, and p-Akt expression was decreased after shikonin treatment compared to the control group in A549/PTX cells. **b** NEAT1 expression was downregulated, and p-Akt expression was decreased in the A549/PTX xenograft. *Statistically significant difference (*p* < 0.05)

NEAT1 has been reported to be associated with paclitaxel-resistance in NSCLC through activation of the Akt signaling pathway (Li et al. 2019). Akt signaling activation results in apoptosis inhibition, cell proliferation, and paclitaxel-resistance in several cancer types (Chen et al. 2018; Zhang et al. 2017b). To observe the effect of shikonin on the expression of NEAT1 and Akt signaling in paclitaxel-resistant NSCLC, qRT-PCR, Western blot analysis and IHC were performed in vitro and in vivo. It showed that the level of NEAT1 expression was downregulated, and p-Akt expression was decreased after shikonin treatment compared to the control group in A549/PTX cells (Fig. 3a). In vivo, it has also been found that NEAT1 expression was downregulated, and p-Akt expression was decreased in the A549/PTX xenograft (Fig. 3b).

### NEAT1 and Akt signaling downregulation was involved in the shikonin against paclitaxel-resistant NSCLC

**Fig. 4:**
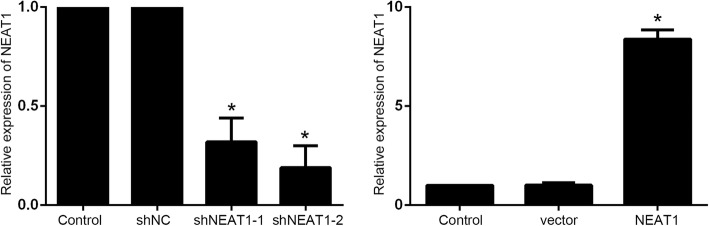
The NEAT1 knockdown and NEAT1 overexpression A549/PTX cells were established. *Statistically significant difference (*p* < 0.05)

**Fig. 5:**
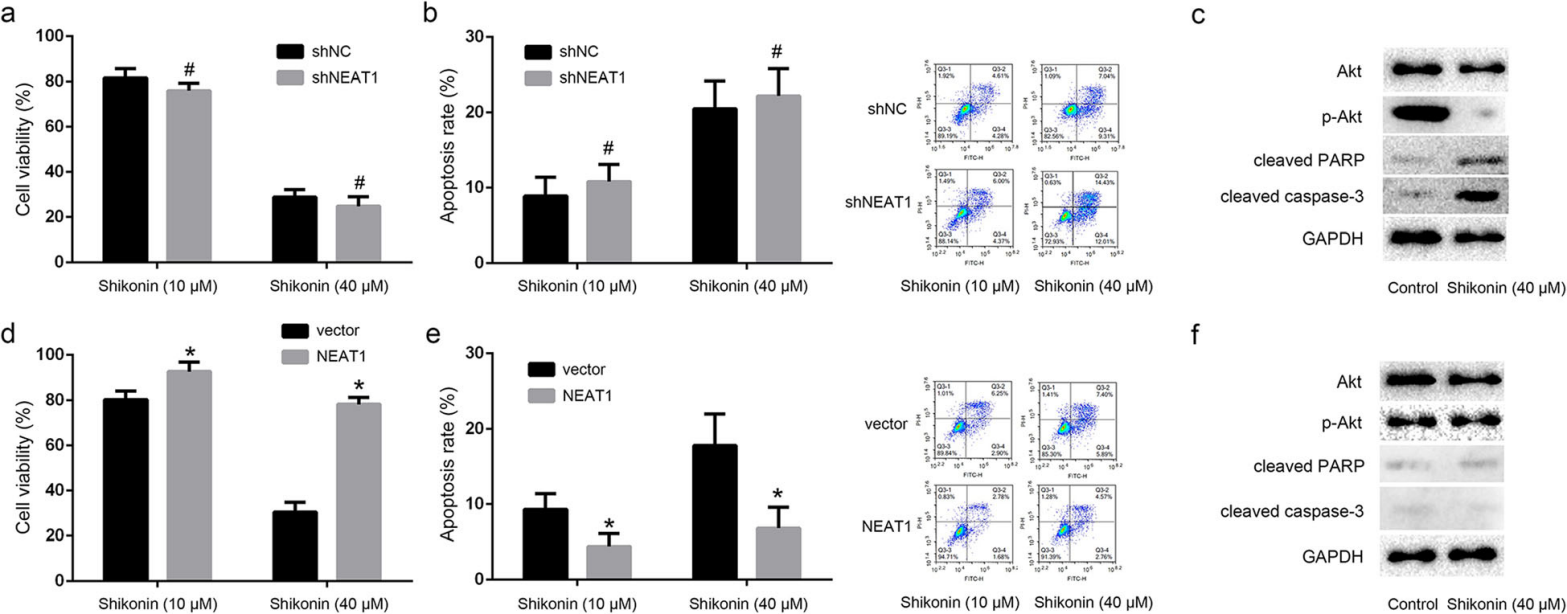
NEAT1 and Akt signaling downregulation were involved in the shikonin against paclitaxel-resistant NSCLC. **a** Either low dose or high dose of shikonin considerably suppressed the NEAT1 knockdown A549/PTX cell growth. **b** Either low dose or high dose of shikonin considerably induced the cell apoptotic death in NEAT1 knockdown A549/PTX cells. **c** p-Akt expression was decreased, cleaved PARP and cleaved caspase-3 expression were increased after shikonin treatment in NEAT1 knockdown A549/PTX cells. **d** The proliferation of NEAT1 overexpression A549/PTX cells was not inhibited after shikonin treatment. **e** Apoptosis rates were not enhanced after shikonin treatment. **f** Shikonin could not decrease p-Akt expression, also could not increase cleaved PARP and cleaved caspase-3 expression after shikonin treatment in NEAT1 knockdown A549/PTX cells. *Statistically significant difference (*p* < 0.05). #No statistically significant difference(*p* > 0.05)

We further established the NEAT1 knockdown and NEAT1 overexpression A549/PTX cells to explore the role of NEAT1/Akt signaling in shikonin against paclitaxel-resistant NSCLC (Fig. 4). The results showed that either low dose or high dose of shikonin considerably suppressed the cell growth and induced the cell apoptotic death in NEAT1 knockdown A549/PTX cells. p-Akt expression was decreased, cleaved PARP and cleaved caspase-3 expression were increased after shikonin treatment (Fig. 5a, b, c). However, the proliferation of NEAT1 overexpression A549/PTX cells was not inhibited and apoptosis rates were not enhanced after shikonin treatment, and shikonin also could not decrease p-Akt expression and increase cleaved PARP and cleaved caspase-3 expression (Fig. 5d, e, f). These results indicated that NEAT1/Akt signaling played an important role in the shikonin against paclitaxel-resistant NSCLC.

## Discussion

Lung cancer is the most common malignancy with poor prognosis and a low survival rate. The development of drug resistance and the lack of therapeutic options are the main obstacle for lung cancer treatment. Finding an effective therapeutic agent without adverse side effects and the acquisition of drug resistance is necessary. Shikonin has been shown to suppress tumor growth by modulating multiple signaling pathways including p21, PI3K, ERK, and p53 (Jeung et al. 2016), and overcome cancer drug resistance such as gefitinib-resistance by inhibiting TrxR and activating the EGFR proteasomal degradation pathway (Li et al. 2017). Shikonin could also enhance the antitumor effect of gefitinib in EGFR wild-type lung cancer via inhibition of PKM2/stat3/cyclinD1 signaling (Tang et al. 2018), and inhibit migration and invasion of lung cancer cells via inhibition of c-Met mediated EMT (Hsieh et al. 2017). It has been reported that shikonin could overcome cisplatin resistance, afatinib resistance, and tamoxifen resistance in several cancer types (Li et al. 2018; Zhang et al. 2017a; Wang et al. 2018d). In the present study, we explored the anti-cancer activity of shikonin on the paclitaxel-resistant NSCLC. In vitro and in vivo study, it has been found that shikonin decreased paclitaxel-resistant NSCLC cell viability and inhibited the growth of xenograft tumor without mice body weight loss. And shikonin markedly induced apoptotic cell death of paclitaxel-resistant NSCLC cells. These results demonstrated that shikonin inhibited paclitaxel-resistant NSCLC through inducing apoptosis.

Phosphoinositide 3-kinase/Akt signaling is dysregulated in most carcinomas and Akt is the key target for cancer therapy. A variety of lncRNAs have been reported to regulate Akt signaling (Dong et al. 2019), and NEAT1 could modulate Akt signaling pathway in several cancers. NEAT1 contributed to radioactive iodine resistance via PI3K/Akt signaling pathway in papillary thyroid carcinoma (Liu et al. 2019). NEAT1 promoted cell proliferation in multiple myeloma by activating PI3K/Akt pathway (Xu et al. 2018). NEAT1 regulated proliferation and invasion of cervical carcinoma by targeting Akt signaling (Guo et al. 2018). NEAT1 promoted the cell growth of prostate cancer through SRC3/IGF1R/Akt pathway (Xiong et al. 2018). NEAT1 impacted cell proliferation and apoptosis of colorectal cancer via regulation of Akt signaling (Peng et al. 2017). Besides, our previous study showed that NEAT1 mediated paclitaxel-resistance of NSCLC through activation of Akt signaling (Li et al. 2019). In the present study, we explored the molecular mechanism of NEAT1 and Akt signaling which was involved in the shikonin against paclitaxel-resistant NSCLC. It has been found that shikonin suppressed the level of NEAT1 and Akt signaling of paclitaxel-resistant NSCLC cell lines and xenograft tumors. The level of NEAT1 expression was downregulated, and p-Akt expression was decreased after shikonin treatment compared to the control group in A549/PTX cells and A549/PTX xenograft. We next established the NEAT1 knockdown and NEAT1 overexpression A549/PTX cells and found that shikonin considerably suppressed the cell growth and induced the cell apoptotic death in NEAT1 knockdown A549/PTX cells, and p-Akt expression was decreased, cleaved PARP and cleaved caspase-3 expression were increased. However, the proliferation of NEAT1 overexpression A549/PTX cells was promoted and apoptosis rates were not enhanced after shikonin treatment, and p-Akt expression was increased. These results indicated that NEAT1/Akt signaling played an important role in the shikonin against paclitaxel-resistant NSCLC.

## Conclusion

Shikonin suppressed the growth of paclitaxel-resistant NSCLC by inducing apoptotic cell death through downregulating the level of NEAT1 and Akt signaling. NEAT1 and Akt signaling downregulation were involved in the shikonin against paclitaxel-resistant NSCLC. Shikonin could be a promising candidate for paclitaxel-resistant NSCLC treatment.

## Data Availability

All data generated or analyzed during this study are included in this published article.

## Abbreviations

lncRNA: long non-coding RNA
NEAT1: Nuclear-enriched abundant transcript 1
NSCLC: Non-small cell lung cancer
PTX: Paclitaxel
